# Multiple Papillomata of the Rectum

**Published:** 1910-02-26

**Authors:** 


### Surgery.

### MULTIPLE PAPILLOMATA OF THE RECTUM.

It occasionally happens that a patient of the cancer age presents himself suffering from symptoms of chronic obstruction of the lower bowel which have come on comparatively recently, have progressed, and have possibly been associated with the passage of a little mucus and blood per rectum, so that it seems at first sight almost certain that he must be suffering from carcinoma of the sigmoid colon or rectum. Occasionally in such cases the physician may be greatly relieved by finding that the symptoms are due not to a malignant but to multiple simple growths of a papillomatous nature, and it may be possible to reach one or more of these with the finger introduced per rectum. The condition cannot be said to be absolutely common, but it is one that occurs sufficiently often to merit more attention than it receives in most textbooks.

The papillomata of which we are speaking may be as much as an inch or more long and of the girth of one's finger. If surgical measures are required it is not a difficult matter to remove such of them as are within the pelvic colon. If the condition should occur in a young person it may be mistaken for colitis or chronic dysentery or tuberculous ulceration of the bowel or ulcerative colitis, but the diagnosis is at once cleared up when one of the papillomata can be reached by rectal examination.

### An Illustrative Case.

Dr. Philip James, surgeon to the Wellington Hospital, New Zealand, reports the following case*: A clerk, aged twenty-eight years, was brought to him by a friend under whose notice he had just come. He was an only child; both parents living, age about sixty years and in good health; grandparents lived to be very old. He himself had never suffered from any illness other than an occasional attack of influenza. There was no history of tuberculosis or other hereditary tendency.

In January 1907 he began to suffer from pain and tenesmus, accompanied by the passage per rectum of clear mucus, or, to use the patient's own words, "like the stuff that comes from the nose." In February diarrhœa set in, ordinary loose motions, not attended by much pain; tenesmus was present. In March blood made its appearance, not mixed with the motion, but at the end of defæcation. He was under medical treatment during these three months, but derived no benefit. With short intervals of quiet, he continued to be very bad until July and August, when he consulted a third medical man, who told him that he had "a nervous breakdown." From this time onwards he continued in much the same state until the end of December 1908, when he consulted yet another doctor, who appears to have been the first to recognise that an examination of the rectum was desirable, and who allowed Dr. James to examine the patient with him. During these two years the patient had become emaciated with an anæmic, cachectic look about him which did not augur well for his future. His weight had become reduced to about 8 st. 5 lb., his normal weight ranging from 10 st. 7 lb. to 10 st. 10 lb.

On January 1, 1909, Dr. James made a digital examination of the rectum, and detected the presence of several large papillomata. Owing to the extreme nervousness and apprehension of the patient he was not able to learn much more with the speculum than that the mucous membrane generally was in a state of intense congestion.

### The Treatment.

The patient was admitted into hospital on January 5. On the following day, under an anæsthetic, the sphincter was dilated and a thorough examination of the rectum made, the previous diagnosis being thereby confirmed. On the right postero-lateral wall were two or three large sessile papillæ about half an inch long and somewhat less in diameter at the base. In addition to these were some smaller ones, scattered about on the posterior wall. Stretching across the posterior wall from the neighbourhood of these papillæ was a well-defined cicatricial band, doubtless the remains of former papillomata which had ulcerated and disappeared. This band led up to another large papilloma on the left lateral wall, which was bleeding freely, and was situated about three inches from the sphincter. The rectal mucous membrane, as far as it could be seen, was deeply congested. At the time of this examination there was no discharge other than the bleeding from the ulcerated papilloma. Two days later the patient was again placed under an anæsthetic, and, the sphincter having been thoroughly dilated by means of a fenestrated pile forceps, the rectal mucous membrane was drawn down as far as possible, and with the thermo-cautery all the papillomata within reach were freely cauterised. A suppository of half a grain of morphia was administered and the patient was returned to bed. From this date he improved, but although the bleeding from the rectum and the tenesmus had ceased, he still continued to have loose offensive stools, with occasional small clots of blood. His appetite, however, was on the increase, and he was clearly putting on flesh.

### After Results.

After about three weeks in hospital he began to be restless and home-sick, and therefore on February 4 he was allowed to go home, on the distinct understanding that he would report himself periodically to his medical attendant; before he left the hospital another rectal examination was made, and the mucous membrane was found to be nearly free from congestion. There was no bleeding, and the cauterised papillæ were gone. He continued to gain ground slowly until about March 15, when his weight was 8 st 12 lb. Hearing then that he was losing ground again, Dr. James advised him to return to the hospital for appendicostomy; this operation finally cured him.

---

* *The New Zealand Medical Journal.* Vol. 7. No 31.

